# Evaluation of an ad hoc paper-based syndromic surveillance system in Ibaraki evacuation centres following the 2011 Great East Japan Earthquake and Tsunami

**DOI:** 10.5365/wpsar.2017.8.3.006

**Published:** 2018-12-20

**Authors:** Matthew M Griffith, Yuichiro Yahata, Fujiko Irie, Hajime Kamiya, Aika Watanabe, Yusuke Kobayashi, Tamano Matsui, Nobuhiko Okabe, Kiyosu Taniguchi, Tomimasa Sunagawa, Kazunori Oishi

**Affiliations:** a Infectious Disease Surveillance Center, National Institute of Infectious Diseases, Tokyo, Japan.; b Ibaraki Prefectural Government, Ibaraki, Japan.; c Field Epidemiology Training Program, National Institute of Infectious Diseases, Tokyo, Japan.; d Kawasaki City Institute for Public Health, Kawasaki, Japan.; e National Mie Hospital, Division of Clinical Research, Mie, Japan.

## Abstract

Outbreaks of infectious diseases can occur after natural disasters as vital services are disrupted and populations move into evacuation centres. National notifiable disease surveillance may be inadequate in these situations because of resource-consuming disease confirmation or system interruptions. Although syndromic surveillance has been used as an alternative in post-disaster situations, no systematic evaluations of it have been published. We evaluated the ad hoc paper-based syndromic surveillance system implemented in evacuation centres in Ibaraki prefecture after the 2011 Great East Japan Earthquake and Tsunami. We assessed the simplicity, acceptability, data quality, timeliness and portability of this system and reviewed its usefulness. We concluded that the system was simple, acceptable, portable and useful. The documentation and monitoring of disease events and trends were useful for developing interventions in evacuation centres and have since been used to improve post-disaster infectious disease and surveillance knowledge in Japan. We believe timeliness was a challenge due to the chain of data transmission and communication passing through an intermediary. Future implementations of this system could consider a more direct chain of data transmission and communication from collectors to analysers. Too few key informant interviewees and the inability to obtain original paper-based data from evacuation centres limited our findings; we conducted this evaluation four years after the response occurred. Future evaluations should be completed closer to when operations cease. The usefulness of the system suggests adopting it in future disasters. A simple, plain-language manual should be developed to improve future employment.

## Introduction

On 11 March 2011, the world’s fourth most powerful earthquake since 1900 (magnitude 9.1), struck north-eastern Japan. (*1*) The earthquake and subsequent tsunami killed 15 894 people and injured 6152, (*2*) and 470 000 were moved into evacuation centres. (*3*) Although the National Epidemiological Surveillance of Infectious Diseases (NESID), Japan’s passive system of sentinel and notifiable-disease reporting, was functional, surveillance staff in the affected areas were drawn into response activities that limited their time for NESID. The Infectious Diseases Surveillance Center (IDSC) in Japan’s National Institute of Infectious Diseases, therefore, designed an ad hoc paper-based syndromic surveillance system in evacuation centres to detect outbreaks among displaced populations.

Syndromic surveillance of symptoms indicative of disease has been used in evacuation centres after previous disasters, (*4*-*7*) although no system has been systematically evaluated. We aimed to evaluate the ad hoc paper-based syndromic surveillance system implemented after the 2011 Great East Japan Earthquake and Tsunami to understand its performance and appropriateness for future disasters and to contribute to post-disaster surveillance knowledge.

## Methods

We conducted this evaluation according to the Centers for Disease Control and Prevention’s (CDC) Updated Guidelines for Evaluating Public Health Surveillance Systems (*8*) four years after the Great East Japan Earthquake and Tsunami had occurred.

### System description

The objectives of the ad hoc paper-based syndromic surveillance system were to: (1) collect daily counts of syndromes of evacuation centre residents; (2) assess daily outbreak risk; (3) and generate timely recommendations to prevent the spread of disease.

IDSC requested that public health nurses and non-health-care staff at evacuation centres record the number of residents presenting with each syndrome (**Table 1**) by age group (< 5 years, 5 to < 65 years and ≥ 65 years) on paper forms and then fax them each day to the local public health centre or prefecture public health department, depending on jurisdictional arrangement. For cases of suspected influenza, public health nurses used rapid influenza kits to test for infection. Positive tests were to be reported as influenza and negative as acute respiratory infection syndrome. A form was to be submitted each day that residents were in the centre, and zero reporting was required. Local public health centres faxed the forms to the prefecture where they were compiled into an electronic spreadsheet and e-mailed to IDSC by 12:00 the following day.

**Table 1:** Definitions for reportable syndromes from evacuation centres following the 11 March 2011, Great East Japan Earthquake and Tsunami, Ibaraki prefecture, Japan, 21 March–15 May 2011

Syndrome	Definition
Influenza	Sudden fever > 38 °C, body pain, cough and sore throat and positive rapid test
Acute gastrointestinal infection	Diarrhoea, vomiting or bloody stool
Acute respiratory infection	Any respiratory system infection symptom, such as cough, sore throat, wheezing, that is not confirmed influenza
Acute neurological infection	Convulsions, difficulty opening mouth, difficulty swallowing or loss of consciousness
Fever with rash	Rash or blisters on the face or body plus fever
Wound-associated infection	Wound with pus or fever
Other	Any other symptom or syndrome

IDSC monitored the data daily for unusual increases and, if detected, would communicate with the prefecture public health department to verify information and discuss response actions. Each week, IDSC also summarized the data, developed histograms for each syndrome, made maps of evacuation centre locations and stratified syndrome counts by municipality and evacuation centre. IDSC used this information, in combination with reported NESID data from surrounding areas, to assess the risk for outbreaks in evacuation centres using the World Health Organization’s (WHO) Communicable Disease Risk Assessment: Protocol for Humanitarian Emergencies. (*9*) Summaries, assessments and recommendations were fed back weekly on electronic slides to the prefecture public health department, which distributed them to local public health and evacuation centres.

### Evaluation description

This evaluation was conducted to assess the system’s sensitivity, data quality, simplicity, acceptability, timeliness, and portability. (*8*, *10*) We could not assess sensitivity without a gold standard or comparative system with which to compare.

Data sources included forms submitted daily to IDSC from the Ibaraki Public Health Department with the numbers of syndromes, evacuation centres, and evacuation centre residents, as well as additional comments; electronic slides sent from IDSC to Ibaraki Public Health; e-mails containing those data and slides; and qualitative information obtained through interviews.

We interviewed a staff member of the Ibaraki Public Health Department who had worked on the surveillance system’s operations and two staff from IDSC: one who oversaw the design and implementation of the system and one who designed and operated the system, analysed its data and developed and disseminated assessments and recommendations. We conducted interviews in November 2015–March 2016.

### Attribute assessment

To assess data quality, we counted the number of missing values in cells where data were expected and expressed that number as a percentage of completeness. This included fields for syndrome counts and the number of evacuees, but not optional cells such as comments. We estimated validity by cleaning the data, counting the number of errors identified and expressing the sum as a percentage of the total number of non-missing values. Errors were defined as values out of the acceptable range or logically inconsistent with other values.

We assessed simplicity by reviewing information flow, case definitions and operating procedures. To assess portability, we reviewed procedural documentation as well as adaptations made to the system and their effects on performance. We assessed acceptability by reviewing prefecture and dissemination reports to determine what percentage conformed with the requirements that (1) a report be submitted each day by 12:00 from the prefecture public health department to IDSC; and (2) dissemination reports were fed back weekly from the IDSC to the prefecture public health department. All three attributes were included in the interviews.

To assess timeliness, we estimated reporting delay by calculating the number of hours between close of business and the time the e-mail containing data was sent from the public health department to IDSC as indicated in the e-mail time stamp and rounded to the closest hour. We then obtained range, interquartile interval (IQI) and median.

We calculated implementation time by counting the number of days, rounded to the nearest whole day, between the date of the disaster and the date of the first report from the public health department, based on the e-mail time stamp. We asked key informants about their perceived timeliness of procedures and implementation.

We reviewed the usefulness of this system by asking interviewees about how the system-generated information was used to prevent disease or improve knowledge. We reviewed trends in reported syndromes to determine if any responses should have been triggered. We analysed quantitative data with Epi Info 7.1.5. (CDC, Atlanta, GA, USA).

## Results

### System implementation

IDSC offered this system to the four most affected prefectures; Ibaraki prefecture was the only one to implement it. Of the others, one experienced massive population emigration, which led to the closure of evacuation centres; one developed a different surveillance system in collaboration with a local university; and one adopted parts of this system late in the post-disaster period but analysed their data internally.

**Fig. 1:**
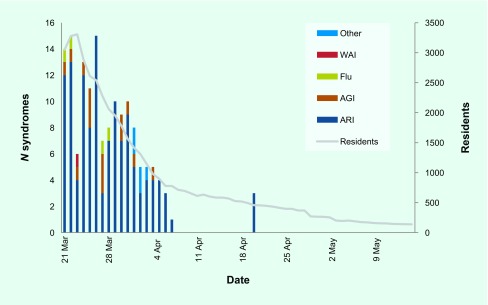
Number of persons identified for each syndrome (*n* = 152) and number of evacuation centre residents, by date of report, following the 11 March 2011, Great East Japan Earthquake and Tsunami, Ibaraki prefecture, Japan, 21 March–15 May 2011

WAI: wound-associated infection; Flu: influenza; AGI: acute gastrointestinal infection; ARI: acute respiratory infection.

Note: no syndromes for fever with rash or acute neurological infection were reported.

There were 95 evacuation centres open in Ibaraki prefecture with residents reaching a single-day maximum of 3305 and minimum of 139. In total, 152 syndromes were reported: 127 acute respiratory infection syndromes, 15 acute gastroenteritis infection syndromes, five “other” without clarification, four influenza and one wound-associated infection (**Fig. 1**).

### Data quality

Among 38 875 expected data cells, 18 665 were missing values (48%), and 403 of the non-missing values contained errors (2.0%). An additional 22 values should have been blank, giving 425 total errors (2.1%).

### Simplicity

Case definitions were in plain language with recognizable symptoms. Syndrome counts were collected at the evacuation centres without investigation, follow-up or laboratory tests (except for suspected influenza that used rapid tests that could only be employed by public health nurses). These counts were recorded each day with a total evacuation centre resident count.

After the third day of system operations, the information flowed through three units only: evacuation centre, prefecture public health department and IDSC. Prior to this, there was an additional reporting unit. In addition, the reporting of all syndromes together and not by age group also changed from day three, which improved the simplicity of the system.

Data were analysed at IDSC by one person using descriptive statistics, histograms and maps. Risk assessments were performed according to an established tool. Interviewees perceived the system to be mostly simple, except that the risk assessments tried to cover too many topics, lacked local context and were not written in plain language.

### Portability

No procedural documentation or manual existed for the surveillance system, yet changes were made to the system without disruption. These included the submission of total syndrome counts only and direct reporting to the public health department instead of through public health centres first.

### Acceptability

The public health department reported to IDSC on 52 of 53 days (98.1%) with seven reports (13.5%) received before the established time. Over eight weeks, seven (87.5%) dissemination reports were fed back. Interviewees revealed all evacuation centres were participating within three days of accepting residents and reported data on most non-holiday weekdays. Interviewees reported that most operators within the system were willing to participate.

### Timelines

**Fig. 2:**
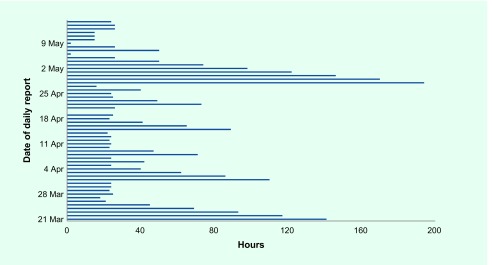
Reporting delay in hours from 18:00 on day of collection to receipt at IDSC, 21 March–15 May 2011

Note: On 20 April, no report was submitted.

The median reporting delay between close of business on the day the data were collected and the time the e-mail containing those data was sent from the public health department to IDSC was 26 hours (IQI: 24–71; range: 2–194) (**Fig. 2**). Implementation time was 10 days after the disaster occurred.

### Usefulness

The system met its objectives: daily counts of syndromes for evacuation centre residents were collected on 52 of 53 days, the daily outbreak risk was assessed and weekly assessments with recommendations were generated. The surveillance system data had no trends that should have triggered a response.

Interviews revealed four usefulness themes: (1) risk assessments could have been more useful for evacuation centre staff by prioritizing syndromes, considering local context and using language more appropriate for non-health care staff; (2) disease trends and risk assessments were valuable for prefecture authorities; (3) dissemination reports were used for developing interventions; and (4) disaster epidemiology knowledge increased since syndrome trends documented by this system have been presented to disaster and medical associations throughout Japan.

## Discussion

We evaluated the ad hoc paper-based syndromic surveillance system implemented in evacuation centres in Ibaraki prefecture, Japan, after the 2011 Great East Japan Earthquake and Tsunami. The straightforward collection, reporting, analysis and feedback procedures of the system made it simple; the influenza testing kits and language used for feedback were the major complications. Interviewee responses, daily reporting and weekly assessments with feedback showed the system’s good acceptability. The simplicity of the system and evidence of adaptation without disruption showed its portability. Finally, the system met its objectives and contributed to situational awareness, interventions and post-disaster surveillance knowledge. Data quality and timeliness were the system’s major challenges.

This is the first published report of a systematic evaluation of a syndromic surveillance system for outbreak detection in evacuation centres following a natural disaster. Similar surveillance system benefits have been identified from other disasters: documenting and monitoring disease events and trends, (*4*-*7*) measuring the burden of disease, (*5*) increasing awareness about reporting, (*4*) dispelling rumours (*4*) and serving as a daily interface with shelter residents. (*7*) Other benefits included measuring the effects of control measures and being timely. (*4*, *5*, *7*)

Timeliness issues may have been due to evacuation centres being operational every day, while the public health department kept its regular hours. The delay in reporting most likely occurred at the public health department since the longest delays occurred on Fridays, Saturdays and during the Golden Week (four national holidays that occur over seven days in late April and early May) when office hours were reduced. These delays improved over time, possibly due to an overworked public health department early on and then an improving post-disaster situation.

Challenges in post-disaster surveillance systems that have been previously published include changing evacuation centre status, (*4*, *5*) competing surveillance systems, (*4*) unstandardized patient recording systems (*4*) and limited coverage. (*4*) In this evaluation, we found that coverage was limited to one prefecture but included all evacuation centres in it. The patient recording systems used were simple and acceptable among participants within the system. There were no changes to evacuation centre status; when a centre closed, the health department reported zero residents.

Data quality was a challenge with low data completeness. Among missing values, > 95% were for missing syndrome counts, potentially because zero reporting was not conducted. There was only one zero reported for a syndrome throughout the period. If missing syndrome counts did mean zero syndromes, then documented counts and trends remain valid; however, there is the possibility that some syndromes were not reported.

Ad hoc paper-based surveillance systems like this one can benefit public health professionals in disaster settings because of their ease of implementation and usefulness during and after the disaster. Fax machines, however, may not be operational in all situations, which may limit the usefulness of this system. Where there is greater destruction, alternative reporting methods may be necessary, such as mobile phone–based applications that have been shown to improve timeliness, although they require careful planning and training before the disaster. (*11*)

Our evaluation was limited by the lack of evidence from evacuation centres, no interviews with evacuation centre staff and no paper-based forms containing the original data. The small convenience sample of interviewees reduced the generalizability of findings, and the long duration between the disaster and interviews may have resulted in recall bias. Interviewees did consult e-mails, notes and other files to improve recall. Future evaluations should be completed soon after operations cease and include representation from all reporting levels of the system. Finally, we were unable to assess the sensitivity of the system because of a lack of comparative information.

For future post-disaster surveillance systems, we recommend that the chain of communication be as direct as possible: preferably, evacuation centre to central command. The public health department should receive the daily summaries but not be directly involved in the system. Removing influenza testing would increase simplicity and avoid needing trained professionals for confirmatory testing, and syndromic data should be sufficient. A manual of operations written in plain language is also recommended, and this should clearly describe zero reporting, the communication of risk assessment findings and the dissemination of reports to non-health care staff. Finally, we recommend pilot testing this system on a mobile phone application.

To conclude, this simple and acceptable ad hoc paper-based surveillance system can be employed quickly and usefully in disaster situations where there are no other options. A simple, plain-language manual should be developed to ensure optimal operation.
